# Cloud-Based Service Information System for Evaluating Quality of Life after Breast Cancer Surgery

**DOI:** 10.1371/journal.pone.0139252

**Published:** 2015-09-30

**Authors:** Hao-Yun Kao, Wen-Hsiung Wu, Tyng-Yeu Liang, King-The Lee, Ming-Feng Hou, Hon-Yi Shi

**Affiliations:** 1 Department of Healthcare Administration and Medical Informatics, Kaohsiung Medical University, Kaohsiung, Taiwan, R.O.C.; 2 Department of Electrical Engineering, National Kaohsiung University of Applied Sciences, Kaohsiung, Taiwan, R.O.C.; 3 Institute of Clinical Medicine, College of Medicine, Kaohsiung Medical University, Kaohsiung, Taiwan, R.O.C.; 4 Cancer Center, Kaohsiung Medical University Hospital, 807 Kaohsiung, Taiwan, R.O.C.; 5 National Sun Yat-Sen University-Kaohsiung Medical University Joint Research Center, 80708 Kaohsiung, Taiwan, R.O.C.; National Health Research Institutes, TAIWAN

## Abstract

**Objective:**

Although recent studies have improved understanding of quality of life (QOL) outcomes of breast conserving surgery, few have used longitudinal data for more than two time points, and few have examined predictors of QOL over two years. Additionally, the longitudinal data analyses in such studies rarely apply the appropriate statistical methodology to control for censoring and inter-correlations arising from repeated measures obtained from the same patient pool. This study evaluated an internet-based system for measuring longitudinal changes in QOL and developed a cloud-based system for managing patients after breast conserving surgery.

**Methods:**

This prospective study analyzed 657 breast cancer patients treated at three tertiary academic hospitals. Related hospital personnel such as surgeons and other healthcare professionals were also interviewed to determine the requirements for an effective cloud-based system for surveying QOL in breast cancer patients. All patients completed the SF-36, Quality of Life Questionnaire (QLQ-C30) and its supplementary breast cancer measure (QLQ-BR23) at baseline, 6 months, 1 year, and 2 years postoperatively. The 95% confidence intervals for differences in responsiveness estimates were derived by bootstrap estimation. Scores derived by these instruments were interpreted by generalized estimating equation before and after surgery.

**Results:**

All breast cancer surgery patients had significantly improved QLQ-C30 and QLQ-BR23 subscale scores throughout the 2-year follow-up period (p<0.05). During the study period, QOL generally had a negative association with advanced age, high Charlson comorbidity index score, tumor stage III or IV, previous chemotherapy, and long post-operative LOS. Conversely, QOL was positively associated with previous radiotherapy and hormone therapy. Additionally, patients with high scores for preoperative QOL tended to have high scores for QLQ-C30, QLQ-BR23 and SF-36 subscales. Based on the results of usability testing, the five constructs were rated on a Likert scale from 1–7 as follows: system usefulness (5.6±1.8), ease of use (5.6±1.5), information quality (5.4±1.4), interface quality (5.5±1.4), and overall satisfaction (5.5±1.6).

**Conclusions:**

The current trend in clinical medicine is applying therapies and interventions that improve QOL. Therefore, a potentially vast amount of internet-based QOL data is available for use in defining patient populations that may benefit from therapeutic intervention. Additionally, before undergoing breast conserving surgery, patients should be advised that their postoperative QOL depends not only on the success of the surgery, but also on their preoperative functional status.

## Introduction

Recent years have seen an increased incidence of breast cancer in Taiwan. Women in the early stages of breast cancer generally have three equally effective surgical options: breast conserving surgery (BCS), modified radical mastectomy (MRM), or mastectomy with reconstruction (TRAM) [1, 2]. However, the recurrence frequency of breast cancer and the number of primary surgical procedures performed for treating breasting cancer have increased in recent years. Therefore, monitoring and analyzing the impact of breast cancer on health outcomes is essential to optimize the use of limited medical resources. Patient-reported quality of life (QOL) is considered an important measure of breast cancer outcomes. Recently, patient-reported QOL has also been used to improve the evaluation of medical treatments and interventions by compensating for the insufficiencies in conventional indicators such as mortality and objective clinical parameters [3, 4].

Although the growing considering of QOL outcomes for breast cancer after surgery, previous studies suffer from several major shortcomings [1–4]. First, none used longitudinal data with more than two time points, and none examined predictors of QOL over a 2-year period. Next, the majority of previous investigations examined populations in economically-advanced countries, where environmental and demographic conditions may differ significantly from poorer regions. Third, appropriate statistical methodologies are rarely used in longitudinal analyses to control for inter-correlations due to repeated measurements within a single patient pool. Finally, although many studies have analyzed QOL outcome data obtained from individual patients, none have proposed a convenient platform for obtaining useful information from the massive amounts of patient-reported QOL data accumulated by healthcare providers from their breast cancer patients.

There are some pros and cons between cloud-based systems and a traditional off-line system. We argued these differences in several perspectives: cost, data process, time and place. First, offline systems generally need to consider the additional spending of personnel and material cost. We need to follow up more times via phone or mail card to guarantee the proper response rate. Conversely, cloud-based system improved the survey efficiency via information communication technology assistance (e.g. e-mail, social media et al.). Second, cloud-based system had less constrain for the data processing than off-line survey (e.g. paper questionnaire). We could get aggregated, integrated, secure data, and downloading from the cloud easily. We did not re-enter data from paper-based questionnaire and reduced the probability of type error. Finally, cloud-based design allows you and your respondent easy access to applications and data from different computers and devices. Meanwhile, cloud applications are browser-based and users are accessible easily from mobile devices, such as tablets and smartphones. Once users register in the cloud, they can access the information from anywhere and anytime. In sum, the cloud-based survey benefits outweighed paper surveys because it ensured wide publication, immediate access, relative speed, and little to no data input or manipulation.

This large-scale prospective cohort study applied generalized estimating equations (GEE) to study QOL changes in a population of women who had received breast cancer surgery. Compared to other prediction methods, GEE is more effective and more precise for predicting QOL changes after breast cancer surgery [1, 2]. A cloud computing service system called the Cloud-based Service Information system of Breast Cancer Patient (*CSIS-BCP*) was then developed to improve the efficiency, agility and usability of survey research, but also to allow healthcare providers and breast cancer patients to easily and conveniently access necessary information. Cloud-computing services offer a distinct advantage in that they allow individuals and organizations to outsource all technology management and maintenance tasks to an expert third party, and to defray expensive equipment purchases, thus providing an extremely cost-effective solution for on-demand services. In the present context, using cloud computing environments also increases accessibility to large amounts of data, thus creating synergies and advantages over conventional methods of investigation and analysis.

## Materials and Methods

### Ethics Statement

This study was approved by the research ethics boards of Kaohsiung Medical University Hospital, Kaohsiung Veterans General Hospital and Chi Mei Medical Center where this study was located. All cloud-based QOL questionnaires were directed by the same two research assistants before and after surgery. The research assistants fully explained the procedures to all of the participants, from whom written informed consent was obtained before enrolling in the study.

### Cloud-based Architecture Design

Quality of life in breast cancer surgery patients was investigated by interviewing related stakeholders such as surgeons, patients, and other healthcare professionals. The interview results were then entered into the *CSIS-BCP* database to support further analysis of QOL in the breast cancer surgery patients.

**Fig 1:**
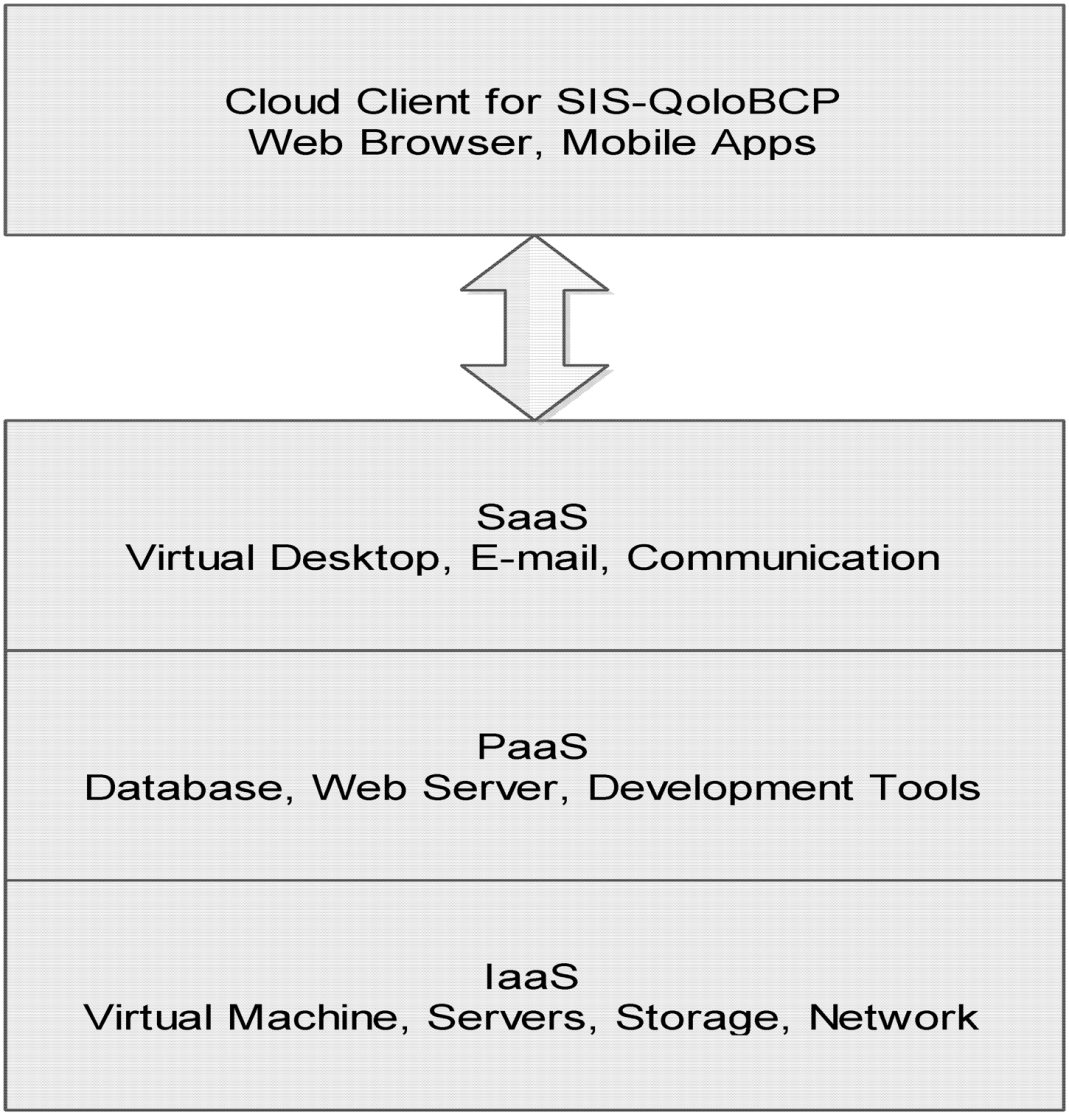
Architecture of the cloud-based Service Information System for Breast Cancer Patients.

Fig 1 shows the three-tier framework of the *CSIS-BCP*. First, the cloud resource manager dynamically deploys virtual machines to process raw data from the respondents. Second, the information extractor retrieves, transforms, clips and merges the processed data from the database management system (DBMS) according to user instructions. Third, the presenter provides a convenient user-friendly interface that enables users to issue query instructions to the *CSIS-BCP* and to view the data retrieved from the cloud system. The *CSIS-BCP* currently supports four main functions. For a specific breast cancer patient, *CSIS-BCP* supports the entry, querying, and display of QOL data. For two or more breast-cancer patients, the *CSIS-BCP* enables comparisons of QOL data [5, 6]. This not only allows researchers to review data retrieved from the DBMS, but also to add new records to the database of the *CSIS-BCP* for analyzing QOL trends in a given patient and comparing QOL with that of other patients.

**Fig 2:**
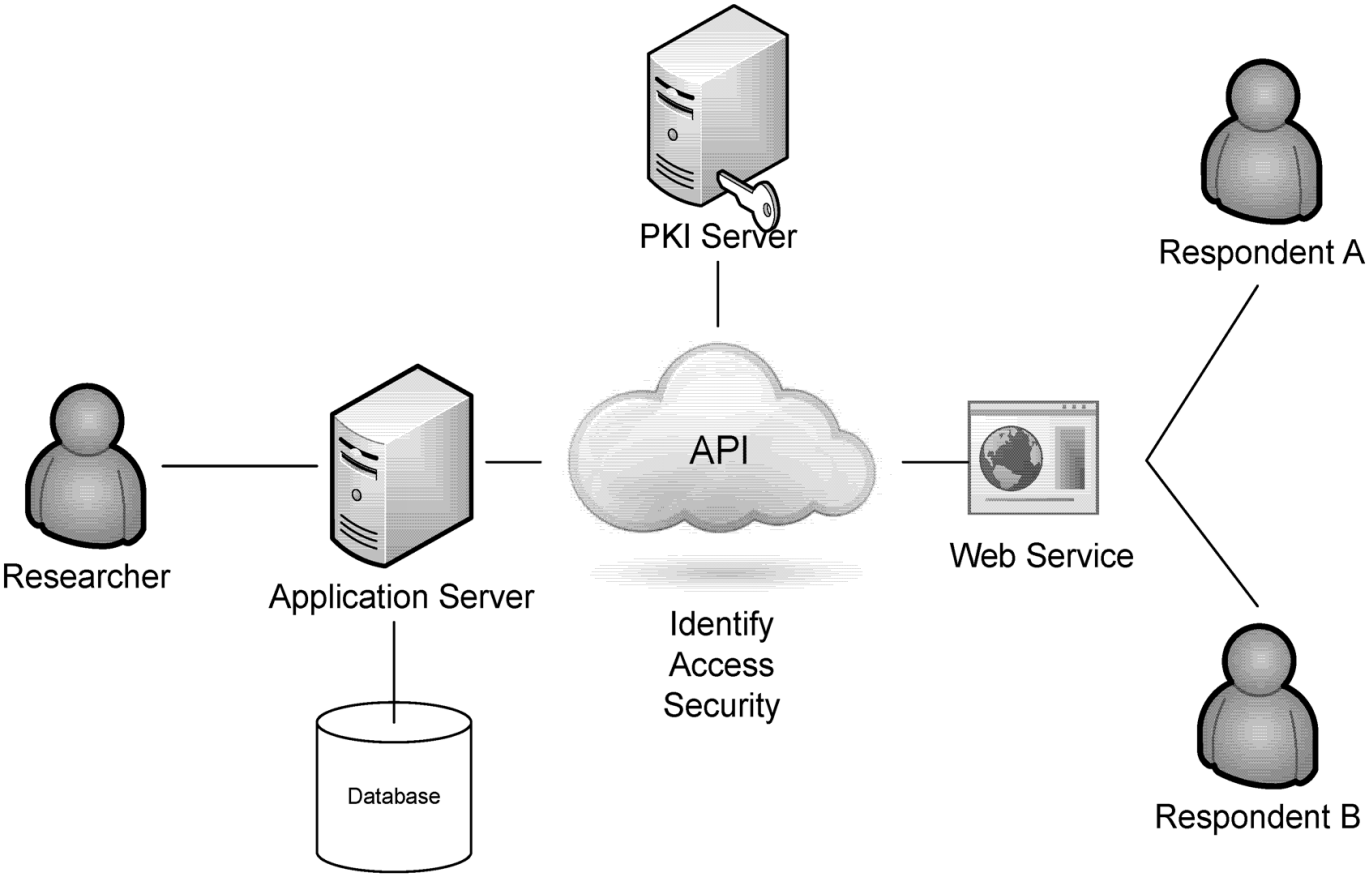
Application processing interface (API) structure.

We designed the application processing interface (API) and it included four major components (Fig 2): application server, database management system, public key infrastructure (PKI), and web-based services [7, 8]. First, the application server provided the user interface and features for researchers and respondents. This study developed different interfaces for the different role of users. Researchers can use the expanded functions such as data management, extract, acquires, and transform the data to the statistical software or predefined processes for statistical analysis. Second, a Database management system was constructed to store the survey data for research, and provided the sources of analysis with our research purposes. In addition, a PKI is the combination of software, encryption technologies, processes, and services that enable this research to secure its communications and data transactions. The ability of a PKI to secure communications and data transactions is based on the exchange of digital certificates between authenticated users and trusted resources. The most secure system was designed in this study to provide the information to qualified researchers, because it restricted under the IRB (KMUH-IRB- 990183) agreements and requirements. Finally, we implement web-based technology to design the user interface with collecting the survey data from target respondents. For example, this study could easily collect the survey data when the respondents were required to enter this system for answering the questionnaires through browser in computer or any devices that connected internet. The researcher login the system via web browser and use the related features, such as extract and transform the survey data for analysis. Certainly, all processes and data transmission were protected under the secured infrastructure. If you need further information about data access, you can contact the corresponding author (Dr. Hon-Yi Shi).

### Study Design and Sample

**Fig 3:**
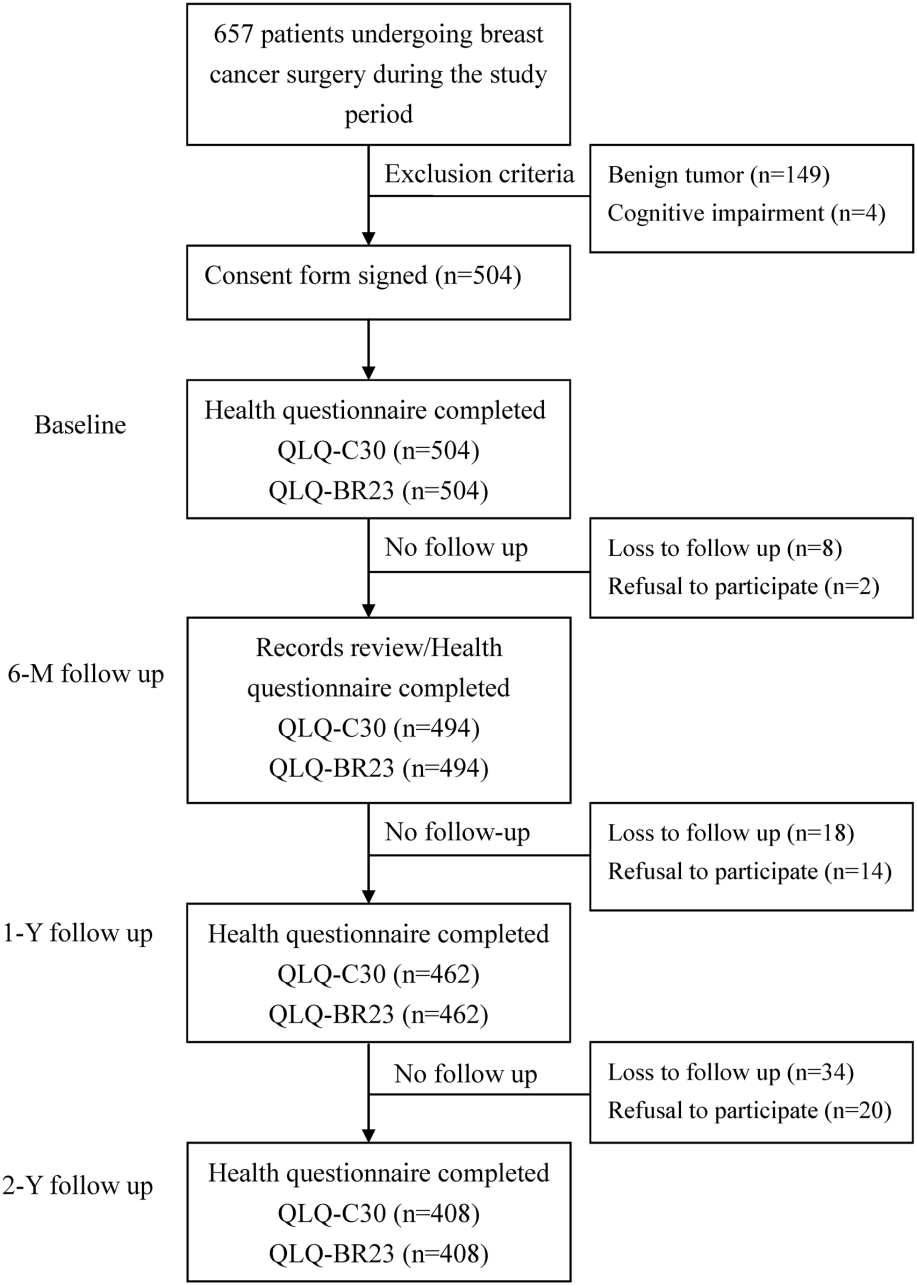
Progression of participants in the trial, including those who met the exclusion criteria, those who withdrew, and those who were lost to follow up.

The sample for this study was taken from a pool of 657 patients with incidental breast cancer and was treated between August 2010 and September 2012 at three medical centers in southern Taiwan. Patients without metastasis were informed of surgical options (BCS, MRM, or TRAM). Patients with benign tumors (n = 149) or cognitive impairments (n = 4), were excluded, leaving 504 patients (Fig 3). Over the following two years, a total of 60 patients were dropped for lack of follow-up, while another 36 were declined to participate further in the study, leaving a total of 408 patients who completed all four surveys.

Prior to surgery, the participating physicians and researchers administered the cloud-based QOL questionnaire survey at the hospital. At 6 months, 1 year, and 2 years after surgery, the participants completed the survey at home or in the hospital during clinical visits. Following implementation of the *CSIS-BCP*, between August and September 2013, 60 stakeholders participated in a system usability assessment.

### Instruments and Measurements

The QLQ-C30 and QLQBR23 questionnaires developed by the European Organization for Research and Treatment of Cancer (EORTC) were used to assess QOL. The EORTC QLQ-C30 is a generic QOL measure for cancer patients which comprises a global health status/QOL subscale, five multi-item functional subscales (physical functioning, role functioning, emotional functioning, cognitive functioning, and social functioning), and several single/multi-item symptomatic subscales (fatigue, pain, nausea and vomiting, dyspnea, insomnia, appetite loss, constipation, diarrhea, and financial difficulties). All Likert scale scores (from 1 to 7 in the global health status subscale and from 1 to 4 in other subscales) were linearly transformed into scores of 0–100 where higher scores indicate better functional status or worse symptomatic problems. The EORTC QLQ-BR23 measures aspects specific to breast cancer and comprises 23 items distributed among four functional subscales (body image, sexual function, sexual enjoyment, and future perception) and five symptomatic subscales (systemic therapy, side effects, breast symptoms, arm symptoms, and upset over hair loss). Chinese versions exist for the EORTC QLQ-C30 and EORTC QLQ-BR23, and these have been successfully used for studies of Taiwanese breast cancer patients [1, 9]. Most of the QLQ-C30 and QLQ-BR23 symptom subscales refer to systematic treatment outcomes. Thus, the scope of the present study is limited to subscales for global quality of life and function.

Many studies have used the SF-36 to profile generic QOL as it allows researchers to compare patients suffering from different diseases in different countries [1, 2]. To provide a useful comparison of overall physical and mental health of study participants against the broader population, we calculate physical component summary (PCS) scores and mental component summary (MCS) as dependent variables using norm-based scoring methods. High SF-36 PCS and MCS subscale scores indicated good QOL.

### Medical Record Review Measures

The following patient data obtained by medical record reviews and questionnaire interviews were tested as independent variables in this study: age, education, living with immediate family, body mass index (BMI), number of pregnancies, breast cancer history, other breast disease history, Charlson co-morbidity index (CCI), tumor stage, surgical procedure, American Society of Anesthesiologists (ASA) class, chemotherapy, radiotherapy, hormone therapy, post-operation length of stay (LOS), post-hospitalization 30-day complications, and preoperative functional status (QLQ-C30, QLQ-BR23, and SF-36 subscales).

### Statistical Analysis

The unit of analysis was the individual breast cancer surgery patient in this study. First, descriptive statistics were tabulated to produce demographic profiles of the patients. Longitudinal data analysis was based on multiple observations, showing high variability between subjects but low variability within subjects. Ordinary regression methods assume observational independence and thus were not well suited for analyzing longitudinal relationships, given this high within-subject correlation.

The GEE approach was used to explore Longitudinal changes in each QOL subscale were examined using GEE and survey data from four time points: prior to surgery, and 6-month, 1-year and 2-year post-op. Each QOL subscale was used as a dependent variable as a function of time and effective covariates, which included age, BMI, average LOS, tumor stage, chemotherapy, radiotherapy, hormone therapy, and preoperative functional status. While similar to a repeated-measures analysis of variance, GEE can compensate for incomplete data at multiple assessment points without creating conflicts with the remaining data, making it particularly useful for dealing with incomplete data in longitudinal studies with continuous outcomes. Autoregressive GEE analysis was used to analyze longitudinal associations when using data for more than two time points.

The recent literature defines usability as effectiveness and efficiency in providing user satisfaction and in assisting the user in achieving his/her specific goals [10]. Here, a usability evaluation was performed to explore the effectiveness of the *CSIS-BCP*. Lewis’ Post-Study System Usability Questionnaire (PSSUQ) was used to assess the information system in terms of user satisfaction and usability [11]. The PSSUQ survey was used to obtain feedback from users who had completed the specific tasks to measure the usefulness, ease of learning, information quality, and interface quality of the evaluation constructs. These questions were answered using a scale from 1 (strongly disagree) to 7 (strongly agree).

In this study, statistical analyses were executed using Stata Statistical Package, Version 10.0 (Stata Corp, College Station, TX). The sample size in this study was sufficient to detect a 5-point difference over time in all QOL subscales, assuming an *α* of 0.05, a power of 80%, and an inter-temporal between-score correlation of 0.80.

## Results

### Descriptive Statistics

Subjects who remained in the study throughout the 2-year period and those who were lost to follow up between the 6^th^ and 24^th^ month after discharge did not statistically differ in terms of age, education, living with immediate family, BMI, number of pregnancies, breast cancer history, other breast disease history, CCI score, tumor stage, surgical procedure, ASA class, chemotherapy, radiotherapy, hormone therapy, post-operation LOS, 30-day post-hospitalization complications, or preoperative functional status.

The mean age of the study population was 52.21 (± 9.59) years (Table 1). On average, 96.03% of the patients lived with their immediate family, and 82.63% had a history of other breast disease. The overall CCI was 0.59 ± 0.99. Of these 504 patients, stave IV metastatic disease was confirmed in 11 patients, including four, three and four with respective lung, liver and bone metastases. Twenty-four patients had a history of BCS before receiving the surgical procedure analyzed in this study.

**Table 1:** Characteristics of the study sample (N = 504).

Variables		Mean ± SD	N (%)
***Patient characteristics***			
Age at operation (years)		52.21 ± 9.59	
Education (years)		9.43 ± 4.58	
Living with immediate family	No		20 (3.97)
	Yes		484 (96.03)
Body mass index (kg/m^2^)		23.85 ± 3.61	
Number of fetuses (cases)		2.35 ± 1.17	
Breast cancer history	No		444 (88.09)
	Yes		60 (11.91)
Other breast disease history	No		416 (82.63)
	Yes		88 (17.37)
Charlson co-morbidity index (scores)		0.59 ± 0.99	
Tumor stage	Stage 0/I		199 (39.45)
	Stage II		184 (36.48)
	Stage III/IV		121 (24.17)
***Hospital Characteristics***			
Surgical procedure	MRM		293 (58.06)
	BCS		141 (28.04)
	TRAM		70 (13.90)
ASA class			
	I		50 (9.93)
	II		390 (77.42)
	III		64 (12.65)
Chemotherapy	No		129 (25.56)
	Yes		375 (74.44)
Radiotherapy	No		290 (57.57)
	Yes		214 (42.43)
Hormone therapy	No		285 (56.57)
	Yes		219 (43.43)
Post-operation length of stay (days)		3.09 ± 1.49	
Post-hospitalization 30 days	No		405 (80.40)
	Yes		99 (19.60)
Complications	No		439 (87.10)
	Yes		65 (12.90)

BCS = breast conserving surgery, MRM = modified radical mastectomy, TRAM = transverse rectus abdominis myocutaneous flap surgery (mastectomy with reconstruction), ASA = American Society of Anesthesiologists.

### Longitudinal Changes in QOL

For each time point, Table 2 shows the mean value, standard error, and p value of each QLQ-C30, QLQ-BR23 and SF-36 subscale for the breast cancer surgery patients after adjusting for patient characteristics and hospital characteristics. In all patients, the QLQ-C30 and QLQ-BR23 subscale scores significantly improved between the preoperative period and the sixth month after discharge (*P* < 0.01) and then remained stable for the rest of the two-year period. When using the sixth month after discharge as a baseline, all QOL subscales other than that for global quality of life were significantly improved by the first year after discharge (*P* < 0.05). Assuming a baseline of first-year scores, all QOL subscale scores were significantly improved except for QLQ-C30 global quality of life, QLQ-BR23 body image, and QLQ-BR23 future perception.

**Table 2:** Mean value and standard error for each QLQ-C30, QLQ-BR23 and SF-36 subscale score measured at varying time points after adjusting for patient characteristics and hospital characteristics.

Subscales	Baseline	6^th^ month	1^st^ year	2^nd^ year
***QLQ-C30***				
Global quality of life	50.06±26.86	71.24±29.58**	77.28±27.59	79.05±30.50
Physical functioning	37.03±15.26	64.57±19.08**	70.48±21.36*	84.44±18.93*
Role functioning	37.32±15.60	60.24±16.87**	69.09±21.80*	86.93±20.79**
Emotional functioning	32.61±12.99	62.42±18.38**	71.80±19.20**	86.31±17.46**
Cognitive functioning	35.48±14.67	63.53±18.07**	73.10±21.26**	89.41±20.79**
Social functioning	64.05±18.40	72.08±17.12*	79.11±20.63*	95.33±21.91**
***QLQ-BR23***				
Body image	33.71±28.93	70.21±24.39**	79.96±30.62*	82.16±32.83
Sexual functioning	53.83±20.49	64.50±16.60**	72.14±24.80*	81.31±26.06*
Sexual enjoyment	45.93±26.72	63.79±14.01**	75.97±22.37**	86.50±33.44*
Future perception	43.30±33.27	65.23±38.28*	80.69±35.36*	86.88±39.98
***SF-36***				
Physical component summary score	44.28±10.28	49.89±10.69**	57.42±14.01**	55.92±16.76
Mental component summary score	42.56±14.10	45.23±12.27*	55.23±13.80**	52.35±14.57

Values are expressed as mean ± standard deviation;

**P < 0.01;

*P < 0.05;

*P* values denote the significance of differences between each time point and baseline after adjusting for patient characteristics and hospital characteristics.

### Results of GEE Models

Table 3 shows the results of multivariate analysis of the effective predictors of QOL in the breast cancer surgery patients. Each time point was significantly related to the QLQ-C30, QLQ-BR23 and SF-36 subscales throughout the 2-year period of the study (*P* < 0.01). After controlling for related variables, breast cancer surgery patients outperformed MRM patients in terms of role functioning, emotional functioning, cognitive functioning, body image and mental component summary, while TRAM patients outperformed BCS patients in terms of physical functioning, emotional functioning, sexual functioning, sexual enjoyment, physical component summary and mental component summary.

**Table 3:** Predictors of each QLQ-C30, QLQ-BR23, and SF-36 subscale after surgery over a 2-year period^#^.

	QLQ-C30						QLQ-BR23				SF-36	
Predictors	GQOL	PF	RF	EF	CF	SF	BI	SF	SE	FP	PCS	MCS
Time												
6^th^ month vs. baseline	14.20**	20.03**	18.32**	24.69**	26.53**	10.05*	23.21**	10.24**	12.79**	17.54**	5.67**	5.07**
1^st^ year vs. baseline	19.04**	32.59**	30.82**	38.80**	36.28**	16.73**	35.43**	18.14**	26.61**	24.39**	9.89**	10.64**`
2^nd^ year vs. baseline	26.59**	46.69**	41.64**	44.01**	40.37**	24.36**	46.15**	19.97**	29.86**	31.36**	12.82**	11.69**
Surgical type												
MRM vs. BCS	-1.49	-1.18	-3.76*	-3.98**	-5.15**	0.74	-5.39**	1.90	-0.20	0.43	-0.46	-2.48*
TRAM vs. BCS	1.36	4.67*	2.62	5.86**	2.98	0.21	-2.95*	3.39*	3.24*	1.06	3.24*	3.49*
Age	-0.23**	-0.03	-0.02	-0.14*	-0.02	-0.04	-0.42*	-0.28**	-0.29**	-0.17*	-0.07	-0.12*
CCI score	-0.07*	-0.08*	-0.08*	-0.10**	-0.09*	-0.03	-0.11**	-0.09*	-0.07*	-0.09*	-0.07*	-0.10**
Tumor stage												
Stage III/IV vs. stage 0/I	-0.12*	-0.24**	-0.22**	-0.28**	-0.30**	-0.11	-0.25**	-0.14*	-0.14*	-0.18*	-0.18*	-0.24**
Stage II vs. stage 0/I	-0.04	-0.06	-0.06	-0.05	-0.09*	-0.02	-0.08*	-0.03	-0.04	-0.04	-0.03	-0.01
Chemotherapy												
Yes vs. no	-4.91*	-4.63*	-0.52	-3.20*	1.32	0.78	-4.10*	-1.68	-1.56	-6.45**	-3.57*	-2.45*
Radiotherapy												
Yes vs. no	3.62*	-1.56	1.63	3.57*	-1.12	2.01	5.29**	2.84	-1.41	4.74*	-1.03	1.03
Hormone therapy												
Yes vs. no	3.68*	0.57	1.68	-0.68	-0.82	0.94	4.52**	2.13	1.75	3.93*	0.34	1.08
Post-operative LOS	-0.03	-0.06*	-0.09**	-0.12**	-0.18**	-0.02	-0.14**	-0.11**	-0.06*	-0.04	-0.07*	-0.08*
Preoperative QOL scores	0.39**	0.50**	0.61**	0.64**	0.57**	0.44**	0.48**	0.31**	0.42**	0.54**	0.48**	0.55**

^#^Values expressed as coefficients.

GQOL = global quality of life, PF = physical functioning, RF = role functioning, EF = emotional functioning, CF = cognitive functioning, SF = social functioning, BI = body image, SF = sexual functioning, SE = sexual enjoyment, FP = future perception, PCS = physical component summary, MCS = mental component summary, BCS = breast conserving surgery, MRM = modified radical mastectomy, TRAM = transverse rectus abdominis myocutaneous flap surgery (mastectomy with reconstruction), CCI = Charlson co-morbidity index, LOS = length of stay, QOL = quality of life.

*P<0.05,

**P<0.01

Furthermore, in relatively older patients, QOL was statistically lower in those with the following characteristics: high CCI score, tumor stage III or IV, previous chemotherapy, and long post-operative LOS (*P* < 0.05). Conversely, patients who had received radiotherapy and hormone therapy generally had better QOL than patients who had not. In addition, patients with high preoperative QOL scores had high scores in all subscales for QLQ-C30, QLQ-BR23 and SF-36.

### Usability Evaluation Results

The PSSUQ results (Table 4) indicated that the participants were satisfied with the usability of the *CSIS-BCP*. The usability scores for specific components of the PSSUQ were as follows: system usefulness, 5.6±1.8; ease of use, 5.6±1.5; information quality, 5.4±1.4; interface quality, 5.5±1.4; and overall satisfaction, 5.5±1.6.

**Table 4:** Measurement items and results of Post-Study System Usability Questionnaire.

Items	Measurement	Results	Construct^#^
1	The system is easy to use	5.90±1.23	System usefulness 5.63±1.78
2	The system helps me to perform research efficiently	5.71±1.45	
3	The system helps me to perform research effectively	5.82±1.42	
4	Learning to operate the system is easy	5.44±1.13	Ease of use 5.60±1.51
5	I understand how to operate the system	5.83±1.71	
6	I can find information I need	5.57±1.25	Information quality 5.41±1.35
7	The information provided by the system is easily understood	5.45±1.12	
8	The information provided by the system helps me to complete projects efficiently	5.76±1.17	
9	Using the system interface is enjoyable	5.59±1.33	Interface quality 5.45±1.37
10	I enjoy using the system interface	5.48±1.45	
11	The system has all the functions and abilities I expected	5.50±1.52	Overall satisfaction 5.50±1.62
12	I am satisfied with the overall system	5.61±1.74	

^#^ System usefulness (items 1–3), ease of use (items 4–5), information quality (items 6–8), interface quality (items 9–10) and overall satisfaction (items 11–12).

## Discussion

Substantial differences were found among the cohorts in terms of age, CCI score, LOS, and tumor stage; however, the striking similarity of the data strongly supports the validity of the results. To examine longitudinal QOL trends, survey information was analyzed using the categorical time variable adjusted for patient and hospital characteristics. Regression model results reveal a significant performance improvement in each QOL subscale in the first 6 months following surgery (*P* < 0.01) and these improvements continued over the following 2 years. These results show that trends for each QOL subscale vary according to the complexity and the involvement of the lower extremities.

Clinical and epidemiological researches increasingly rely on cloud-based patient-reported data, usually obtained by questionnaires, responses to which are often combined into final scores. Statistical strategies used to analyze such data are usually selected based on classical test theory (CTT) instead of item response theory (IRT) due to researchers being more familiar with CTT. While CTT uses observed scores that can be presumed to accurately represent “true” scores, IRT uses an underlying response model that relates the responses to a latent parameter. Therefore, further work is needed to assess the suitability of one or both methods in longitudinal questionnaire data analysis.

The BCS group scored higher than the MRM group for role functioning, emotional functioning, cognitive functioning and body image but the TRAM group produced significantly larger subjective improvements in terms of physical functioning, emotional functioning, sexual functioning and sexual enjoyment. This may be due to the relatively younger age and earlier stages of the tumors of patients in this group.

Age is also found to be an independent predictor of breast cancer surgery outcomes, reflecting findings that older patients experience greater improvements in QOL subscales than younger patients [1, 2]. While older patients have more co-morbidities and less social support, GEE models control for the number of co-morbidities and the observed improvement in health outcomes may reflect selection bias in that physicians may apply selection criteria based on patient and hospital attributes associated with increased likelihood of improvement. In addition, BCS focuses on improving body image and sexual function, thus younger patients are more likely to experience optimal health outcomes.

Many co-morbidities are found to be linked with low QOL subscale scores, and previous reports have found that many co-morbidities are strongly associated with poor post-operative functional status [12, 13].

Patients in the present study were found to have QLQ-BR23 scores for body image, sexual functioning, and future perspectives, comparable to the Dutch and American samples studied by Sprangers et al., but lower scores for sexual enjoyment [14]. Compared with the results of a study in Iran, however, our patients were found to have better scores for body image and future perspectives than those reported in Iran [15], but lower scores for sexual functioning, and similar scores for sexual enjoyment. This variation may be due to cultural changes as Taiwan’s has modernized over the past few decades, adopting more western values. Different follow-up time points and sample sizes could also contribute to the discrepancy. Also, breast cancer surgery women in Taiwan are more focused on survival and thus pay less attention to their body image and sex life.

A final interesting finding is that, over the 2-year period of the study, the single best predictor in each QOL subscale was preoperative health status, which is consistent with reports that the best predictors of postoperative outcomes are preoperative QOL scores. As this issue has been addressed elsewhere, data from a randomized trial comparing early and delayed breast cancer surgery would be of great value to clinicians when selecting the optimal timing of surgery. Therefore, effective counseling is essential for apprising patients of expected post-surgery impairments. If QOL outcomes are considered benchmarks, then accurately assessing preoperative functional status (a major predictor of postoperative outcome) is crucial.

Cloud computing technologies offer many advantages over conventional information technology infrastructure, including support for on-demand access to massive computational resources and massively reduced costs to the user. Therefore, implementing the *CSIS-BCP* in a cloud environment enhances its flexibility, accessibility and usability [9–12], and our experimental results confirmed that the *CSIS-BCP* indeed enables researchers to integrate and analyze questionnaire data for breast-cancer patients easily and efficiently.

Other objectives of this study were to clarify the actual requirements of the *CSIS-BCP*, to solve privacy issues and to integrate diverse information from different stakeholders. For an improved interpretation of the data obtained in this study, eight post-interviews were performed at 1 year after the initial survey with the researchers, physicians, other caregivers, and patients. All interviewed participants agreed that the survey results provided insights into the successful use of a cloud-based technology that integrates the results of surveys of breast cancer surgery patients. However, the interviews also revealed that a major concern was that the user interface could not be customized for different types of users. Another issue raised was whether widespread adoption of the *CSIS-BCP* would be cost-effective. Therefore, further studies are needed to develop and evaluate the intangible value of the *CSIS-BCP* to provide a cloud-based service that is adaptable to changing researcher needs in the turbulent cancer research environment.

This is the first and a pilot study for designing a cloud-based system. We proposed to create the unique study environment in private cloud for the following reasons: First, the study data will be protected seriously under the ethical issues and regulations in Taiwan. Privacy concerns became the necessary consideration to protect the research data and respondents in the system development, such as referring the Health Information Technology for Economic and Clinical Health Act to secure electronic health information exchange or follow similar regulations in Taiwan. Second, informed consent forms for research studies now are required to include extensive detail on how the participant's protected health information will be kept private. Therefore, we build these mechanisms on the private cloud environment for on-premises and on-demand scalability. Base on previous discussions, we did not select the commercial cloud service (e.g. Amazon EC2, Google Cloud, etc.) not only commercial product has security and information disclosure concerns, but also to consider the policy issues and related regulations with compliance in Taiwan. Furthermore, private cloud services allow this study more control over the cloud infrastructure, improving security and flexibility, because the user and the network are subject to special restrictions for maintaining privacy, and will not share personal information without express consent.

We also provide research purpose and process in detail before the survey. Inform sense addressed and accepted respondent’s agreement, the system applied tokenization to provide a user id and password. For technical reasons, an encryption scheme usually uses a pseudo-random encryption key generated by an algorithm, and Tokenization applied to protect the privacy issues in this study. Hence, all symmetric cryptographic operations are based on AES-128 in our system. Generate privacy concerns when using an additional virtual server management, in order to avoid data theft. Although mega.co.nz similar commercial products can be used, but considering the application of the disclosure to commercial sites. Besides, commercial products are due to be attacked and to user and password-based private wastage have doubts. This study may refer the HIPAA proposed scheme in the future.

Additionally, this study conducted the cost assessment that cloud-based compared to off-line (paper-based) methods. Generally, offline systems generally need to consider the additional expenditure like the previous comments discussion. Each respondent takes the traditional manner, the total costs than the cloud-based survey system is 4 to 5 times higher, and got the low response rate. The cloud-based system could provide the on-demand scalability and meet research requirements or scale in the future. In sum, this study found whether in tangible and intangible benefits, this system is superior to the traditional off-line method of investigation.

Although all research questions were adequately and satisfactorily addressed, several limitations are noted. First, this study collected data from breast cancer surgery patients under the supervision of three surgeons in three different medical centers, each of whom had the highest surgeon volume in their respective hospitals. Such a sample selection procedure ensures that the limited experience of a surgeon does not significantly influence patient outcomes. By focusing the analysis on patients treated by these three orthopedists, the results of this study are thus more representative of all breast cancer surgery patients compared to one analyzing patients treated by a single surgeon. However, a notable limitation is that, in the prospective patient cohort, the first patient was enrolled in 2007. Therefore, depending on their inclusion date, some surveyed patients experienced longer follow-up than others, which may have caused selection bias. Nonetheless, subjects who remained in the study throughout the 2-year period and those who were lost to follow up between the 1st and 2nd years after discharge did not statistically differ in patient characteristics, hospital characteristics, or in any of the aforementioned preoperative QOL parameters (data not shown).

Current trends in clinical medicine emphasize therapies and interventions to improve QOL, and patients more likely to benefit from interventions could potentially be identified through analyzing cloud-based QOL data. In addition to the surgical procedure, several other factors should be accounted for when evaluating post-operative QOL. Surgeons can use pre- and post-operative consultations to raise the awareness of breast cancer surgery candidates regarding the expected course of recovery and functional outcomes, and should to explain that pre-operative functional status has an important impact on postoperative QOL.
